# Our Journal

**Published:** 1871-07

**Authors:** 


### PART IV.

### Editorials, Reviews, Items and News.

### OUR JOURNAL.

THE GEORGIA MEDICAL COMPANION was started as the representative of the best and loftiest principles of the profession. It will not be conducted upon any exclusive line, or for selfish purposes and objects. It is the Journal of no clique, but is an independent worker, applauding without hesitation the "good, beautiful and true" in the profession everywhere. It bases all its actions and utterances upon truth—truth, as it shimmers along the pathway of principle and law. It breathes no spirit of persecution, indulges in no ebulitions of hatred and harbors no resentments in view of the unceasing provocations to pervert it from its holy purposes.

With these principles inscribed upon its banner, it has won the respect and received the support of the purest and best men of the profession of the State. From every section words of cheer and encouragement greet it, and bid it God-speed upon its mission of vindicating the truth, upholding the imperishable principles of the profession, and of contributing its quota of medical truths to swell the onward flow of science.

In order to accomplish our highest aims, and to subserve these great interests, we have, in carrying out our own wishes, and to meet the suggestions of others, secured the co-operation of some of the first medical minds of the State as Asssociate Editors.

In the accomplishment of this purpose, the profession will have the united energies of all these gentlemen in making the COMPANION the exponent and reflex of the principles and interest of the entire profession of the State, and now, more than ever, will zealously advocate the principles upon which it is founded, and become an earnest worker in the delightful fields of our time-honored science.

With the aid of these gentlemen and their friends, we will be able to make our Journal, in all of its departments, everything that the profession could expect or desire. Our Original Department will be open to the profession, through which they may commune one with another, upon questions of Science, and through which the active workers of our "art divine" may become the instruments of love and mercy to our people.

In our second and third departments, we will strive to cull and glean from every quarter the cream of medical literature, bearing upon every subject legitimately connected with our science for the alleviation and prevention of disease. In these departments we hope to gather, by the aid of all, the many gems of thought and useful formulæ in the possession of our professional brethren for the common good; making them the repositories of sparkling gems, and practical thoughts, condensed from the medical literature of the past and present.

We invite every true friend of the profession to aid in this good work. We ask them to co-operate with us, and the gentlemen who are associated with us; to give us the benefit of their counsels and assist us in giving an impetus to scientific research and investigation in our State; to build up a Journal which shall truly represent the sentiments of the profession, and make it a garner for the use of all. We desire to follow, not to lead; to become a co-worker with every member of the profession, in every section of the State, for the promotion and advancement of a common calling and heritage. Suggestions having these noble purposes for their object, will be cordially received. Anything and everything looking to the advancement of our science; to the collective and individual promotion of every member of the profession, will be our pleasure to advocate and support. With these objects in view, we ask the active co-operation of all, as a means of making our Journal the friend and exponent of Science, and a common heritage for an honored brotherhood.

We do not purpose to make it the medium through which any shall indulge in personal feeling. But it will advocate the rights of all—the humblest—when professionally assailed, or in their efforts to uphold the honor and dignity of the profession.

With these "words" we make a "new departure" on the highway of professional Journalism, again asking the aid and patronage of all true friends of science, that we may honorably acquit ourselves, and that our Publisher may enjoy the material support he so justly deserves in his efforts to serve the profession.

